# Social Media Depiction of Cleft Lip and Cleft Palate: Instagram Versus YouTube Shorts Analysis: Instagram Post Versus Instagram Reel Analysis

**DOI:** 10.3390/cmtr18010004

**Published:** 2025-01-03

**Authors:** Joshua Lewis, Manav Patel, Nangah Tabukumm, Wei-Chen Lee

**Affiliations:** 1 John Sealy School of Medicine, University of Texas Medical Branch, 301 University Boulevard, Galveston, TX 77555, USAmanmpate@utmb.edu (M.P.); nntabuku@utmb.edu (N.T.); 2 Department of Family Medicine, University of Texas Medical Branch, 301 University Boulevard, Galveston, TX 77555, USA

**Keywords:** cleft lip, cleft palate, social media, Instagram, YouTube Shorts

## Abstract

Study Design: Qualitative analysis study. Introduction: Social media has been pivotal in the dissemination of medical knowledge to the public. The aim was to identify the demographics of individuals posting about cleft lip and palate on YouTube Shorts and Instagram, to characterize the content of these posts, and to highlight factors that could aid surgeons in better educating patients with cleft lip and palate. Methods: Instagram posts and YouTube Shorts with “#cleftlip”, “#cleftawareness”, “#cleftpalate”, “#cleftplipandpalate”, and “#cleftproud” were searched on 8 June 2024. Postings were subclassified and analyzed for content, including topics of posts, authors, media type, tone of the post, and year of post. Results: A total of 3321 posts were analyzed, with 2698 coming from Instagram and 623 from YouTube Shorts. The majority of content creators were patients and their family members (*n* = 2054, 61.8%), cleft lip and palate foundations (*n* = 384, 11.6%), and companies (*n* = 381, 11.5%). Only 167 posts were authored by physicians (5.1%). Among the educational and informational posts, patients and family members accounted for the majority of the posts (409 posts, 57.7%). Physicians contributed to a small fraction of the educational content (37 posts, 5.2%). Conclusions: Physician participation in the cleft lip and palate social media realm on Instagram and YouTube Shorts was found to be limited. Moreover, there was a scarcity of educational content on both platforms, indicating a significant opportunity for physicians to engage more actively in cleft lip and palate social media discussions.

## 1. Introduction

Social media platforms, such as Instagram and YouTube, are constantly adapting to meet the demands and preferences of users, allowing users to connect with specific audiences [1]. The healthcare community has increasingly embraced social media as a platform for patient education and engagement [2,3]. This development has enhanced the communication between physicians, their peers, the general public, and potential patients [4,5,6]. Additionally, social media has increased patient access to health education [7,8]. For example, during the COVID-19 pandemic, various board-certified plastic surgeons, hospitals, academic institutions, and organizations increased their usage of social media to disseminate health-related information [6,9,10]. In addition to the increased output of health information on social media, the type of posting (videos vs. text vs. photos) has correlated with the amount of engagement the posting received. Despite the recognized benefits of social media, healthcare providers’ use of social media remains relatively low, indicating an opportunity for healthcare providers to increase their engagement with patients [5,10,11].

Disparities in cleft lip and palate exist based on gender, race and ethnicity, and socioeconomic status [12,13,14]. The reasons for these significant gaps in cleft lip and palate surgical interventions are multifaceted, potentially including factors such as understanding the impact of cleft lip and palate, lack of knowledge about the benefits of cleft lip and palate repairs, and lack of resources. Previous studies have analyzed the effect of the dissemination of cleft lip and palate education on patients and their parents [15]. Hakim et al. found that increased educational engagement positively correlated with enhanced knowledge and support among parents of children with cleft lip and palate [15]. Social media can serve as a potential resource in the dissemination of information about cleft lip and palate to underserved or vulnerable populations [16,17,18].

Considering the healthcare disparities that exist in cleft lip and palate, as well as the potential for patient education and outreach through social media platforms, increased participation by surgeons in online communities could significantly enhance awareness among both patients and healthcare professionals. A study by Sycinska-Dziarnowska et al. analyzed hashtags (#) and engagement in cleft lip and palate postings [19]. Another study analyzing cleft lip and cleft palate on social media looked at the number of postings, associated hashtags, the number of likes, and who was posting [20]. However, no studies have yet analyzed both YouTube Shorts and Instagram posts in a comparative analysis.

In this study, we investigated Instagram and YouTube Shorts posts related to cleft lip and palate. We analyzed these posts for various attributes, including the author, topic or theme, surgical timeframe, and portrayal of cleft lip and palate, and further categorized the results. The objectives of this study were to understand the demographic posting about cleft lip and palate on these platforms, to describe the content of the posts, and to identify factors that could enhance surgeons’ communication and education efforts with patients with cleft lip and palate.

## 2. Methods

### 2.1. Study Design

This qualitative analysis utilized postings from two widely used social media outlets: Instagram and YouTube Shorts. In our study, we concentrated on Instagram and YouTube Shorts due to their high levels of user engagement and popularity across various demographics. These platforms are known as short-form content platforms. Because the platforms emphasize bite-sized content, they are particularly effective for reaching a broader audience as they have higher shareability and watch time. Thus, these platforms were chosen as most people find these platforms more accessible to watch. Other social media platforms were excluded based on specific criteria such as data accessibility, content type, user engagement and reach, and overall relevance to the community. Instagram may include pictures, texts, or videos, while YouTube Shorts are videos of short duration (either 15, 30, 60, or 90 s). This study analyzed six distinct hashtags: #cleftlipandpalate, #cleftlip, #cleftpalate, #cleftstrong, #cleftproud, and #cleftsmile. We reviewed all posts from both platforms on June 8, 2024 due to the algorithm potentially changing daily. This study focused on posts made between 9 June 2020 and 8 June 2024. Our research study adheres to the ethical principles outlined in the Declaration of Helsinki—a cornerstone document established by the World Medical Association in 1964 to guide medical research involving human subjects. This declaration emphasizes the importance of informed consent, ethical review, risk–benefit assessment, and respect for participant privacy and confidentiality. By following these principles, we commit to ensuring the dignity, rights, and well-being of all participants throughout the course of our study.

### 2.2. Exclusion and Inclusion Criteria

All postings featuring or discussing cleft lip and cleft palate were considered for inclusion. Posts were excluded from both platforms if they were hard to understand, appeared to be generated by automated “bots”, were about non-human subjects, or were not in the English language, as it was unlikely the hashtags would be used in these instances. Additionally, our study only analyzed public posts on both platforms because public posts are readily accessible without the need for special permissions or approvals from individual users or the platform itself. The decision not to include private accounts in our analysis was primarily made to uphold ethical considerations and respect individuals’ privacy preferences.

### 2.3. Primary Outcome and Secondary Outcome

The primary outcome in this study is to assess demographics behind the cleft lip and palate content on social media platforms, specifically Instagram and YouTube Shorts. This includes an analysis of the types of posts, their tone, the settings or timings of the posts, and the authorship. The secondary outcomes examined include the engagement metrics of the posts, such as the number of likes, and the popularity of specific hashtags related to cleft lip and palate. This study also compares content, sources, and engagement between Instagram posts and YouTube Shorts, as well as the distribution of posts across various topics and their tone, whether positive, negative, or neutral. These outcomes aim to provide a comprehensive understanding of how cleft lip and palate are represented on social media and the level of engagement such posts receive.

### 2.4. Analysis

The measures assessed in this study are outlined below. The data collection process was conducted independently by two reviewers (JL and NT) and analyzed using Microsoft Excel 2021 (Microsoft Corporation, Redmond, WA, USA). Prior to commencing this study, a data collection guideline was established to ensure consistency and minimize discrepancies (Table 1). While formulating the data collection guidelines, the two reviewers underwent a process in which they examined examples of posts representing each category. This examination was conducted in the presence of a third reviewer, whose role was to address any discrepancies that arose during the initial stages. This collaborative effort aimed to establish a common understanding of the classification criteria among the two reviewers.

### 2.5. Author Identity

The authors of the posts in this study included patients, their parents/caregivers or family members, physicians, companies, hospitals and academic institutions, foundations (example: Operation Smile), schools, and others. The categorization of post authors was primarily determined through a detailed analysis of the post’s content and caption. For example, a posting discussing the pre-operative and post-operative procedure performed by a plastic surgeon would be categorized under physician. In cases with unclear authorship, authorship was determined by reviewing the profile description and other related postings of the user. Typically, examining the profile description and related posts by the user provided sufficient information to clarify the author’s identity.

### 2.6. Hashtags

This study analyzed six distinct hashtags: #cleftlipandpalate, #cleftlip, #cleftpalate, #cleftstrong, #cleftproud, and #cleftsmile.

### 2.7. Topics of Post

The topics/subjects of the posts were classified into several categories, as detailed in Table 2. The authors determined these categories by examining the content, media, and captions of the posts. For example, a post from a physician featuring a caption and image focused on promoting a product, company, or service were categorized as advertisements. These were distinguished from purely informational or educational posts, which might be shared by a physician or company but were not intended to market their services or products.

### 2.8. Setting/Timing of Posts

The posting setting was categorized as pre-operative, post-operative, or non-operative. Non-operative postings included educational content, questions about cleft lip and palate, or posts from foundations concerning cleft lip and palate.

### 2.9. Tone of Posts

The overall tone of each cleft lip and palate posting was categorized as positive, negative, or neutral. To determine the tone, researchers looked at the media content (picture, video, or text) in addition to the caption. For example, a posting expressing negative emotions about cleft lip and palate surgery would be categorized as negative, whereas a posting promoting the benefits of cleft lip and palate surgery would be categorized as positive. In cases of uncertainty regarding the tone, the posting was classified as neutral.

### 2.10. Statistical Methods

This study tracked the engagement metrics, such as posting likes and the number of followers for each author. These metrics were utilized to gauge popularity across both Instagram and YouTube Shorts. Both social media algorithms assess a range of user engagement indicators to evaluate the popularity and significance of a post. These indicators include likes, comments, shares, saves, and the duration users spend engaging with a post. Posts that receive higher engagement are perceived as more relevant and popular by the algorithm. Additionally, a two-sample t-test and descriptive statistical analysis was conducted to compare differences in sources and content between posts and reels. Statistical significance was deemed at *p* < 0.05.

## 3. Results

Our final analysis comprised 3321 public social media posts, after excluding 1224 posts that fell under the exclusion criteria outlined in Methods section. Instagram contributed the majority of posts (*n* = 2698, 81.2%), while YouTube Shorts contributed fewer posts (*n* = 623, 18.8%) (Table 3).

### 3.1. Media Type

Overall, 62.2% (*n* = 2067) of the posts analyzed in this study were images, 34.0% (*n* = 1128) were videos, and 3.8% (*n* = 126) were text images. Text images were images that contained only text on the posts. Notably, all of the text images came from Instagram. Additionally, all the YouTube Shorts posts consisted of videos (623 posts, 100%), reflecting the platform’s limitations. In contrast, majority of the Instagram posts comprised images (2067, 76.6%).

### 3.2. Author

Family members and patients were the primary authors of cleft lip- and palate-related social media posts (2054 posts, 62.2%), followed by foundations (384, 11.6%), and companies (381, 11.5%). These three author categories collectively accounted for 85.3% of all the posts analyzed in this study. Only a small fraction of the posts was authored by physicians (167 posts, 5.1%) and schools (23 posts, 0.6%). None of the analyzed posts were accounted to midlevel providers. These patterns in authorship were similar between platforms, with patients and family members being the predominant authors on Shorts (517 posts, 83.0%) and Instagram (1537, 57.1%). For YouTube Shorts, patients and family members accounted for nearly all of the posts.

**Figure 1:**
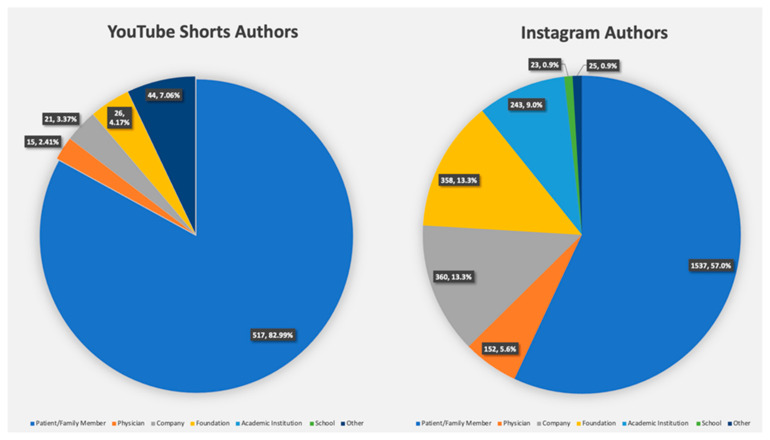
Social media post authors: pie charts depicting author breakdowns for Instagram and YouTube Shorts.

Among the 167 physician posts analyzed, 152 were on Instagram and 15 were on YouTube Shorts, representing only 5.6% and 2.4% of the total posts on each social media platform, respectively (Figure 1). Of the 15 YouTube Shorts posts, 5 were authored by maxillofacial and oral surgeons, and 10 were authored by board-certified plastic surgeons. Also, all the YouTube Shorts posts by physicians were focused on educational content. In total, 152 physicians were identified on Instagram. Of those 152 physicians on Instagram, 33 were maxillofacial and oral surgeons, and 119 were board-certified plastic surgeons. The predominant themes for these posts were advertisement (130 posts; approximately 85.5%), with 22 posts categorized as educational/informational (14.5%). All 243 posts attributed to hospitals or academic institutions were attributed on Instagram (Figure 1).

### 3.3. Topic

**Figure 2:**
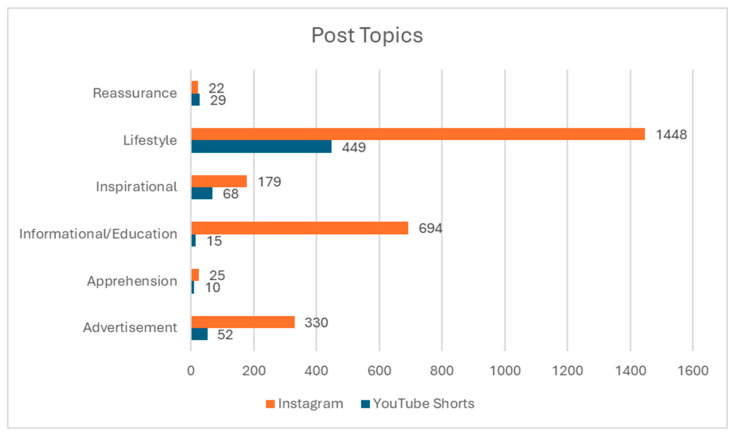
Topic distribution across social media platforms: A bar graph illustrates the prevalence of different topics. Lifestyle content emerged as the most prevalent overall (1897, approximately 57.1%).

Four main topics comprised the majority of posts: lifestyle (1897; approximately 49.9%), informational/educational content (709, 21.3%), advertisement (382, 11.5%), and inspirational (247, 7.4%). Both YouTube Shorts and Instagram featured a significant number of lifestyle posts (1448 on Instagram and 449 on YouTube Shorts). However, out of the 709 educational posts examined, 694 were found on Instagram, and 330 out of 382 posts were also found on Instagram. Only a small fraction of YouTube Shorts videos was educational (15, 2.4%). Additionally, a majority of the educational posts were authored by patients and their family members (409 posts, approximately 57.7%). Physicians accounted for a small portion of all the educational posts on both platforms (37 posts, 5.2%). Figure 2 highlights the distribution of educational posts across the two platforms.

### 3.4. Setting

**Figure 3:**
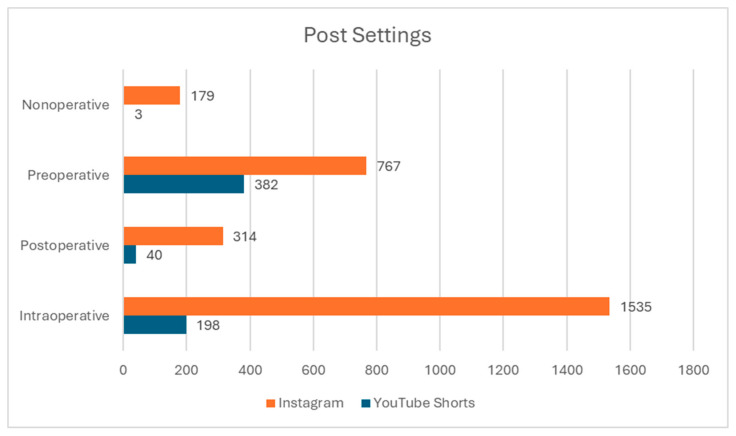
Setting of social media post settings: A bar graph representing the distribution of social media posts based on their setting or timing in post.

The predominant setting or timing for posts was non-operative (1733 posts, approximately 52.2%), with the majority of the remaining posts being post-operative (1149 posts, approximately 34.6%). The distribution varied between platforms: YouTube Shorts had predominantly post-operative posts (382 posts, approximately 61.3%), while the Instagram posts were predominantly non-operative (1535, approximately 57.0%). Figure 3 illustrates the distribution of social media posts based on their setting or timing, highlighting the differences between platforms, with Instagram favoring non-operative content and YouTube Shorts focusing more on post-operative content.

### 3.5. Tone of Posts

**Figure 4:**
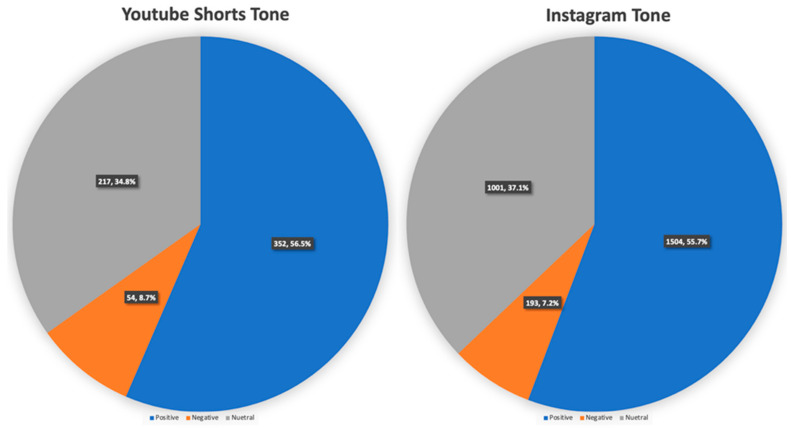
Tone of cleft lip and palate on social media: YouTube Shorts and Instagram. Both platforms predominantly depicted positive tones on their posts.

The majority of social media posts in this analysis portrayed cleft lip and palate in a positive light (1856 posts, 55.9%), with a small number being negative (247 posts, 7.4%) and the remainder being neutral (1218, 36.7%) (Figure 4). This distribution was similar between both social media platforms. Among the negative posts identified, a significant portion was authored by patients and their family members during the post-operative period (56 posts, 2.7%). These posts commonly highlighted the negative aspects of lifestyle (28 posts), expressed concerns about the surgery (11 posts), or discussed surgical complications (17 posts).

### 3.6. Popularity

Among all the Instagram and YouTube Shorts posts examined, the average number of likes was 2782.5. However, these figures were skewed toward higher values, as indicated by a median of 65.4 likes per post. A small subset of posts received thousands of likes, with the highest reaching 75,948, contributing to the elevated mean number of likes in the dataset. Subgroup analysis was conducted on posts with 1000 likes or more, comprising 537 posts (approximately 16.2% of the total). The majority of these highly liked posts were authored by patients and family members themselves (285 posts; around 53.1%), with only 15 post attributed to a company (2.8%) and none to physicians. Furthermore, lifestyle emerged as the predominant topic in these posts (433 posts; about 80.6%). All the highly liked posts were categorized as either post-operative (212 posts, approximately 39.5%) or non-operative (325, approximately 60.5%). It is important to note that many of these posts were created by users known for consistently receiving high numbers of likes on their content, often unrelated to cleft lip and palate.

There was a notable difference in the average number of likes per post between YouTube Shorts and Instagram. YouTube Shorts posts exhibited an average of 4386.6 likes per post, whereas Instagram posts exhibited an average of 167.2 likes per post (*p* = 0.003). Notably, YouTube Shorts had a higher proportion of highly liked posts (*n* = 202, approximately 32.4%) compared to Instagram (*n* = 122, approximately 4.5%). It is worth noting that high-popularity posts on both platforms shared similarities in terms of authorship, setting, topic, and content portrayal.

### 3.7. Analysis of Hashtags

The popularity of hashtags varied across categories, with #cleftlip appearing in 1896 postings, #cleftlipandpalate in 1174, #cleftproud in 1042, #cleftsmile in 542, #cleftstrong in 361, and #cleftpalate in 399 postings. Of note, many posts contained multiple hashtags. An additional analysis showed that #cleftlip, #cleftlipandpalate, and #cleftproud were heavily used in the postings of physicians (*n* = 76, 45.5%). The hashtag #cleftproud was predominantly used by patients and their family members (*n* = 854, 41.6%). Lastly, postings using the hashtags #cleftsmile with #cleftproud had a higher mean number of likes (4534.2) than those employing either hashtag alone (856.5; *p* < 0.01) (Table 4).

## 4. Discussion

This study investigated cleft lip and palate portrayal on Instagram by examining posts and reels for content type, authorship, and engagement. Overall, patients and family members of patients with cleft lip and palate made the most postings, followed by cleft lip and palate foundations. Only 5.1% of the postings were made by physicians, highlighting a need for more engagement from medical professionals on both platforms. Additionally, approximately half of posting (4.9%) analyzed fell into the “lifestyle” category, often portraying patients in their non-operative phase, followed by informational/educational content posts. However, only 5.2% of the educational posts were authored by physicians, indicating opportunities for more informed health communication in cleft lip and palate information on social media.

Our results, which demonstrate that most of the YouTube Shorts and Instagram posts were made by patients and family members, underscore that family members’ and patients’ experiences play a significant role in raising awareness about cleft lip and palate. This category was created because the majority of cleft lip and palate posts were about infants and authored by their parents or another family member. Additionally, some of these posts, although written by the parents, used first-person captions as if the patient with cleft lip and palate was speaking. Parent contributions provide support and information from a personal perspective, which can be invaluable for those undergoing similar experiences with cleft lip and palate. On the other hand, the limited presence of educational content by physicians represents a critical area for improvement. Considering their reach and influence, these platforms represent an untapped resource by which to disseminate accurate medical information and guide patients and families. Additionally, having more educational postings by medical professionals can enhance public understanding of cleft conditions, treatment options, and post-operative care, contributing positively to the overall goal of improving patient outcomes and awareness.

When utilizing social media to discuss sensitive health topics like cleft lip and palate, it is vital to consider ethical principles and prioritize patient confidentiality. While patients and their families often share personal stories and images to create awareness, healthcare professionals and organizations must navigate these platforms cautiously. Posting patient photos, videos, or stories without explicit consent can lead to legal and ethical violations, potentially damaging trust between patients and providers. Compliance with HIPAA guidelines is critical for ensuring patient privacy when using social media for healthcare promotion. These rules protect against the unauthorized disclosure of identifiable patient information, minimizing the risk of privacy breaches that could harm patients and undermine the credibility of healthcare professionals. Developing robust consent procedures and institutional policies can further support ethical practices in sharing patient-related content online. Additionally, the inclusion of well-curated educational materials that adhere to ethical standards can combat misinformation while offering reliable information to patients and their families. Addressing these challenges effectively can improve the credibility and influence of healthcare providers on social platforms.

Protecting patient confidentiality also helps build trust and supports meaningful engagement on social media. Physicians should be mindful when sharing insights about their clinical work or patient-related experiences in public forums. Anonymized case details, general observations, or collaborations with established organizations can serve as appropriate alternatives to directly sharing specific patient information. Social media platforms like Instagram, while being excellent tools for raising awareness, require careful use to ensure the accurate and respectful representation of health conditions. Establishing best practices for healthcare providers—such as verifying the accuracy of shared information, maintaining professionalism, complying with HIPAA standards, and avoiding sensationalism—can enhance both public education and patient empowerment. Collaborations with organizations such as cleft lip and palate foundations can amplify reliable messaging and lessen the individual burden on clinicians to sustain an active and impactful online presence.

The low prevalence of physicians seen on social media posting about cleft lip and palate can be attributed to the numerous challenges they must navigate. Ensuring patient confidentiality is paramount, requiring clinicians to avoid sharing identifiable patient information without explicit consent to prevent breaches of legal and ethical guidelines [21]. Maintaining a professional online presence is also essential, as inappropriate or unprofessional posts can harm a clinician’s reputation and erode public trust [22]. Additionally, clinicians must ensure the accuracy of the information they share, as posting inaccurate or misleading content can have serious implications for patient care and public health [5,23]. Engaging with patients on social media can blur the lines of professional boundaries. Clinicians should establish clear guidelines for online interactions to prevent conflicts of interest and maintain professional integrity [24]. Active engagement on social media can be time consuming, so clinicians must balance their online presence with their clinical responsibilities to avoid compromising patient care. By remaining aware of these challenges and adhering to ethical guidelines, clinicians can effectively utilize social media to improve patient education, engagement, and support while upholding their professional standards.

Social media is easily accessible, and it greatly affects how its users make decisions about most aspects of their life, including their health [25,26]. Social media allows individuals to access a vast amount of information about health concerns, such as cleft lip and palate. In 2020, Cinar et al. showed that parents of infants with cleft lip and/or palate used Facebook to gain more information about the condition [27]. Social media platforms are Internet spaces permitting both misinformation and an ability to create rapport with the public through content creation [28]. Physicians should understand both the reliability of health information and the potential for misinformation on the Internet, as patients are likely to come across both in their search for answers.

This study is not the first to analyze Instagram posts and cleft lip and/or palate. Sycinska-Dziarnowska et al. conducted a study in 2020 to analyze Instagram posts tagged with #cleftlip [19]. Their aim was to look at the expressions associated with primary posts and their replies. Their findings align with ours, in that we also found overwhelmingly positive portrayals of cleft lip and palate posts with certain hashtags. However, no previous studies have analyzed the user demographics, engagement metrics, and potential opportunities for physicians to create rapport with and educate the broad population.

Social media plays an important role in many people’s lives, with its popularity increasing. Patients with cleft lip and/or palate experience a unique journey, full of ups and downs that may not always find expression during regular medical appointments. Additionally, the journey of many parents with their infants with cleft lip/palate is also unique. Social media helps both patients and parents connect with others and with their doctors on a more personal level. Social media can also allow patients and their family members to learn from others worldwide who are going through the same condition. This study found many patients with cleft lip/palate, their families, and foundations sharing their stores on social media. These posters used the hashtags #cleftsmile and #cleftproud to share personal stories on Instagram. To better understand the experience of living with cleft lip and palate, doctors could follow these accounts.

While social media offers numerous benefits, it also comes with significant risks. One major downside of social media is its open accessibility, which means that the identity of authors cannot always be verified. The lack of “gatekeeping” means that postings do not need to be true or accurate or even contain current information. This lack of strict regulation on most social media platforms creates a serious risk of encountering misinformation [4]. Therefore, all social media users must approach online content with caution and verify the information from reliable sources. Additionally, physicians must recognize the need for their presence on social media.

Going forward, physicians have the unique ability to bridge the gap in the understanding of cleft lip and palate between surgeons and the public, by educating about the condition and sharing reliable information on social media. The Centers for Disease Control and Prevention has created instructions to help healthcare providers present unbiased and non-discriminatory messages through social media [29]. These instructions emphasize the significance of healthcare providers using social media platforms to communicate healthcare procedures and information. Additionally, we believe that organizations encompassing physicians should also develop formal guidelines for social media use. Such guidelines will allow physicians who may be hesitant to engage on social media to increase their engagement.

## 5. Limitations

This study faces several inherent limitations, notably its utilization of a sample of non-randomized social media posts, which constrains the interpretation of data to the sampled posts’ population. Those who post on Instagram may self-select as having more of certain characteristics (e.g., more openness to sharing information, greater access to the Internet) than the affected population as a whole. The interpretation of the findings is confined to the specific postings examined. The authors focused their investigation on a substantial subset of posts from Instagram and YouTube Shorts, enabling a thorough analysis of these platforms but restricting this study’s applicability to other widely used platforms like TikTok and Facebook. Although we were able to compare two prominent social media platforms, YouTube Shorts and Instagram, we were unable to extend our analysis to include TikTok due to the TikTok ban in Texas and the absence of approval from the Institutional Review Board (IRB) to undertake a TikTok analysis. Future research should compare video content related to cleft lip and palate on social media platforms including Snapchat, Tik Tok, Twitter, and Facebook to offer a more comprehensive evaluation of cleft lip and palate across social media. Moreover, employing advanced technologies such as artificial intelligence or coding could enhance post analysis, an approach beyond the scope of this study.

Furthermore, this study’s reliance on publicly accessible social media posts excludes insights from private accounts or users who limit their audience due to privacy concerns, a common practice among individuals. Another limitation concerns the nature of YouTube Shorts as a solely video platform; this may influence engagement in ways that Instagram may not. This difference may explain the high like average seen for YouTube Shorts relative to that for Instagram posts. Thus, we could not fully assess whether this imbalance was caused by the algorithm of the two separate platforms or by the nature of the content. Another limitation lies in the subjective nature of data collection, particularly in categorizing posts as positive, negative, or neutral, which may introduce bias and variability in interpretations. The authors showed reliability in categorizing the postings as negative, positive, or neutral. However, other researchers may differ in their interpretation of tone, as this assessment is somewhat subjective.

## 6. Conclusions

Given the potential benefits of active social media engagement, the authors recommend increased participation from oral and maxillofacial surgeons, plastic surgeons, and other related healthcare professionals, such as pediatricians, speech-language pathologists, orthodontists, and primary care physicians, who collaboratively provide comprehensive care for patients with cleft lip and palate. This study provides a basis for practitioners to strategically develop their social media content. This study sheds light on the portrayal of cleft lip and palate on social media, revealing a prevalence of patient-generated content and a scarcity of educational posts from physicians. This finding indicates a potential untapped avenue for physicians to gain significant engagement.

Our findings emphasize the potential of social media to bridge gaps between surgeons and patients, though challenges like misinformation persist. Moving forward, it is essential for healthcare providers to engage responsibly on social media, adhering to ethical guidelines and contributing to the dissemination of accurate information. Further research is warranted to explore social media’s role in patient education and communication, with a focus on addressing the limitations of this study and developing formal guidelines for healthcare providers’ social media use in plastic surgery. Ultimately, leveraging social media responsibly can enhance patient care, advocacy, and connectivity in healthcare.

## Figures and Tables

**Table 1:** Cleft lip and palate social media analysis data collection guidelines.

Data Category	Short Description
Platform	Instagram or YouTube Shorts
Date of post	Exact date of post; format: month, day, year
Author	Patient, parent/caregiver, family member, physicians, companies, hospitals and academic institutions, foundations, schools, other
Media type	Picture, video, or text
Popularity	Based on number of likes for the posting and number of followers of the author
Setting	Pre-operative, post-operative, or non-operative (includes posts by foundations, relaying information, or by businesses)
Topic	Advertisements, apprehension, complication, educational/informational, inspirational, lifestyle, reassurance, other
Tone of the post	Negative, positive, or neutral (used for postings not clearly positive or negative)
Search term	#hashtag used on both Instagram and YouTube Shorts

**Table 2:** Social media posting topics: general categories of cleft lip and palate social media postings.

Topic Category	Definition	Example
Lifestyle	Illustrates the impact of cleft lip and palate on an individual’s daily life	“A posting showing a patient engaging in various daily activities with cleft palate or cleft lip”
Educational/ Informational	Purpose is to inform the public or other healthcare workers about educational material for cleft lip or information about cleft lip	“Cleft lip and palate happen if the tissue that makes up the roof of the mouth between 6–9 weeks of pregnancy doesn’t join together completely.”
Inspirational	Delivers positive and motivating messages concerning the journey and outcomes of cleft lip and palate	“Your journey with cleft palate is unique, but remember, you’re never alone. Your courage inspires us all.”
Reassurance	Acts as a reassuring message within the community about cleft lip and palate surgery	“Today marks a significant milestone! Feeling a mix of nerves and excitement as I embark on this journey. Grateful for the support. Let’s hope for positive outcomes!”
Advertisement	Promotion of a product or service to patients with cleft lip or palate by physicians, hospitals, or businesses	“Our company specializes in speech therapy program tailored specifically for individuals with cleft palate”
Other	Any posting that does not fit into any categories. Other postings were determined by agreement by both reviewers.	

**Table 3:** Summary of social media data: obtained from social media search.

	*n* (%)			*p* Value
Source	Shorts	Instagram	Total	
Included	623 (18.8%)	2698 (81.2%)	3321	0.001
Excluded	262	849	1224	
Media type				0.032
Image	0	2067 (76.6)	2067 (62.2)	
Video	623 (100)	505 (18.7)	1128 (34.0)	
Text	0 (0)	126 (4.7)	126 (3.8)	
Authorship				<0.001
Patient or family member	517 (83.0)	1537 (57.1)	2054 (61.8)	
Physician	15 (2.4)	152 (5.6)	167 (5.1)	
Company	21 (3.4)	360 (13.3)	381 (11.5)	
Foundation	26 (4.1)	358 (13.2)	384 (11.6)	
Academic institution	0 (0)	243 (9.0)	243 (7.3)	
School	0 (0)	23 (0.8)	23 (0.6)	
Other	44 (7.1)	25 (1.0)	69 (2.1)	
Topic				0.002
Advertisement	52 (8.3)	330 (12.3)	382 (11.5)	
Apprehension	10 (1.6)	25 (0.9)	35 (1.1)	
Informational/educational	15 (2.4)	694 (25.7)	709 (21.3)	
Inspirational	68 (10.9)	179 (6.6)	247 (7.4)	
Lifestyle	449 (72.1)	1448 (53.7)	1897 (57.1)	
Reassurance	29 (4.7)	22 (0.8)	51 (1.6)	
Setting				0.001
Non-operative	198 (31.8)	1535 (57.0)	1733 (52.2)	
Pre-operative	40 (6.4)	314 (11.6)	354 (10.7)	
Post-operative	382 (61.3)	767 (28.4)	1149 (34.6)	
Intraoperative	3 (0.5)	82 (3.0)	85 (2.6)	
Tone of the posting				0.001
Positive	352 (56.5)	1504 (55.8)	1856 (55.9)	
Negative	54 (8.7)	193 (7.2)	247 (7.4)	
Neutral	217 (34.8)	1001 (37.1)	1218 (36.7)	
Popularity				0.003
Mean likes per posting	4386.6	167.2	2782.5	
Median likes per post	193	22.4	65.4	

Of 4545 posts, 3321 were included in the final analysis from both platforms.

**Table 4:** Number of posts by hashtags.

Hashtag	Total Number of Posts in Which it Appeared	%
#cleftlip	1896	57.1
#cleftlipandpalate	1174	35.4
#cleftproud	1042	31.4
#cleftsmile	542	16.3
#cleftstrong	361	10.9
#cleftpalate	399	12.0

## Data Availability

The dataset analyzed during the current study is not publicly available due to privacy restrictions imposed by Instagram’s terms of service. Access to Instagram data requires user consent and adherence to platform policies. Researchers interested in replicating or verifying the findings presented in this study can contact the corresponding author for guidance on accessing similar datasets from authorized sources or for assistance in understanding the methodology used.
